# Challenges and Opportunities: Building a Relationship Between a Department of Biomedical Engineering and a Medical School

**DOI:** 10.1007/s10439-016-1785-1

**Published:** 2017-01-09

**Authors:** Steven C. George, M. Elizabeth Meyerand, On behalf of the Council of Chairs of Biomedical Engineering

**Affiliations:** 1 Washington University in St. Louis, St. Louis, MO USA; 2 University of Wisconsin-Madison, Madison, WI USA

**Keywords:** Academic department, Research program, Teaching program

## Abstract

A department of biomedical engineering can significantly enhance the impact of their research and training programs if a productive relationship with a medical school can be established. In order to develop such a relationship, significant hurdles must be overcome. This editorial summarizes some of the major challenges and opportunities for a department of biomedical engineering as they seek to build or enhance a relationship with a medical school. The ideas were formulated by engaging the collective wisdom from the Council of Chairs of the biomedical engineering departments.

## Introduction

A department of biomedical engineering engages in original research to solve important problems in biology and medicine and also the education and training of future biomedical engineers at all levels (undergraduate, masters, doctoral, post-doctoral). It is thus natural to consider professional schools in the health sciences as partners in these endeavors. The benefits of developing a relationship with a medical school, or other health sciences school (e.g., Dental School), are many and include enhancing the clinical relevance and translational potential of the research, access to patients and clinical samples, resources such as unique core labs and revenue streams, access to research space for faculty growth, exposure of students to a clinical environment, broad exposure to NIH-style extramural funding, and philanthropic opportunities to develop significant resources for new or existing initiatives.

Nonetheless, departments must overcome significant obstacles to achieve these benefits including, but not limited to, differences in the types of trainees, salary structure (e.g., 9-month vs. 12-month appointments), criteria for promotion and tenure; teaching expectations; administrative structure; and financial priorities—clinical revenue vs. research. Furthermore, some of the benefits are also of keen interest to other departments in engineering, and a department of biomedical engineering should therefore develop a leadership role that is mutually productive for all of the stakeholders. In an effort to organize and focus current ideas related to these interactions, the Council of Chairs of Biomedical Engineering, consisting of nearly 120 chairs of departments of biomedical engineering across the country, has developed the following document describing “challenges and opportunities” for how to build and maintain a mutually beneficial relationship with a medical school.

While this editorial focuses on challenges and opportunities between a department of biomedical engineering (also known as bioengineering) and a school of medicine, many of these practices are germane to relationships with other professional schools in the health sciences including schools of dentistry, optometry, nursing, veterinary medicine, and public health.

### Tools

Data on current practices and ideas on how to improve relationships between departments of biomedical engineering and medical schools was gathered through two mechanisms: (1) an online survey, and (2) a small group discussion.

#### Survey

**Figure 1:**
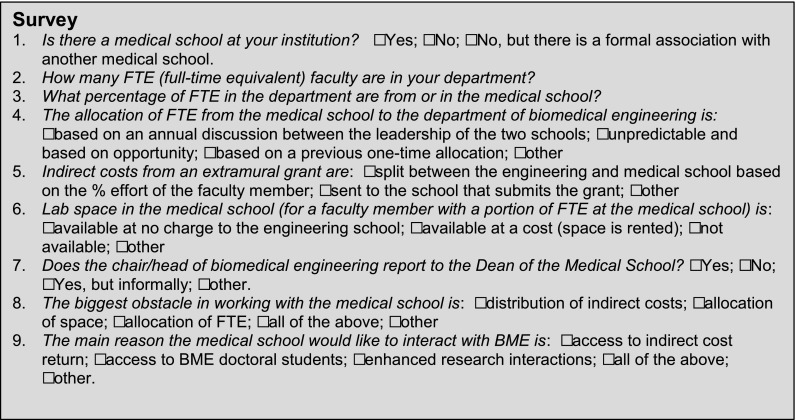
Survey questions provided to the Chairs of the Departments of Biomedical Engineering.

A 9-question survey (Fig. 1) was developed and sent to all of the department chairs on Sept 1, 2015, with a response deadline of September 10, 2015. Fifty-four (54) responses were received representing approximately 50% of the departments. The questions were focused on obtaining some quantitative data on the departments, characterizing current practices, and identifying current obstacles.

#### Small Group Discussion

During the annual meeting of the Council of Chairs, at the annual meeting of the Biomedical Engineering Society (October 7, 2015, Tampa Bay, FL), the results of the survey were presented, followed by a break-out session in which the chairs were divided into six groups of approximately 8–10 people. Each group was asked to discuss the content of the current editorial and identify challenges and opportunities for how to create a productive relationship with a medical school.

## Results

Some broad conclusions can be drawn from the quantitative data of the survey. First, the relationship between biomedical engineering departments and medical schools varies widely as evidenced by the wide range in the number of biomedical faculty that reside in a medical school. Second, the allocation of faculty lines from the medical school into biomedical engineering is generally sporadic, unpredictable, or non-existent. This can be attributed, in part, to the regular turnover in leadership at the school level (Deans) and reflects the importance of maintaining strong relationships between the Dean of Engineering and Dean of Medicine, and an overall commitment at the level of the Provost. Third, there is no single formula for success when developing a strategy to distribute indirect costs or allocate research space; however, a general set of recommendations can maximize the chances of success (see below). Finally, allocating research space and faculty positions are common obstacles, which can be overcome with clear communication and an administrative structure built to facilitate mutually beneficial interactions between schools that have common interests.

Below, we provide the specific recommendations based on the findings of the survey and the small group discussion, which are grouped into four areas: (1) faculty appointments; (2) indirect costs; (3) research space; and (4) events to stimulate interactions.

### Faculty Appointments

Appointing biomedical engineering faculty in the medical school (or *vice versa*) can stimulate communication and collaboration. The appointments can range from complete (100% of the salary fully paid by the medical school), to partial (e.g., 33% medical school, 67% engineering), to courtesy (0% salary) depending on many important factors including, but not limited to: (1) level of faculty member (e.g., Assistant Professor); (2) formal training of the faculty member, (3) an ability to teach engineering courses and/or advise doctoral students; (4) clinical skills and responsibilities; (5) research program focus, and (6) the medical school department. While there is no single set of rules that should govern these appointments, below is a general list of recommendations.

- Pursue split appointments with BME faculty and medical school departments in which both the faculty member and the enhanced interaction with the medical school are beneficial.
- Develop a document that describes details of how the split appointment will function including space, teaching, service, and indirect costs. This document can be in the form of a memo of understanding (MOU) and will encourage consistency across appointments and clarity of responsibilities.
- Tenure and promotion should be the responsibility of a single department to avoid confusion over potentially different standards. This responsibility should be articulated clearly as early as possible to avoid conflicts of commitment.
- Special attention should be taken in describing the salary structure as most medical schools operate with 12-month appointments and engineering schools with 9-month appointments. For example, a faculty member who has a 50:50 appointment may receive 4.5 months of salary from engineering and 6 months of salary the medical school. The remaining 1.5 months of salary represents 50% of the traditional “summer salary” in engineering. In this scenario, the medical school is actually paying a larger portion of the salary, but expectations for the faculty member to pay some or all of this salary from extramural grants may be different than expectations in engineering. When a faculty member receives extramural funding for salary, it should be clear in the MOU how this resource is to be allocated.
- “Courtesy” (or 0% salary) appointments should be linked to a minimal level of activity to ensure engagement, and should be reviewed on a regular basis (e.g., every 3 years). This discourages faculty from simply collecting titles without engaging. However, the review process for re-appoint of these faculty should be streamlined to avoid a significant administrative burden.

### Indirect Costs

Formulas for utilizing indirect costs from extramural grants take a form that is specific to a given university. However, a portion of this resource generally flows to the school that administers the grant, and these costs can be used to cover expenses associated with maintaining the research infrastructure. In some cases a portion of this resource also flows to the department of the faculty member as well as institutes and centers which may play a role. As with faculty appointments, there is no single model for success, but the following recommendations should enhance the likelihood of long-term success:

- A significant portion of indirect costs should flow to the school, department, or center that is responsible for paying for the maintenance of the faculty member’s lab space.
- A portion of the indirect costs should flow to the unit that bears the burden to administer the grant. However, the distribution formula should not place departments and centers/institutes in competition for administering a grant.
- Department faculty members should be provided guidance on how to appropriately balance effort and grant submissions between centers, institutes, and the department that is consistent with the model for success at each campus.

### Research Space

Assigning space to conduct research should not be complicated, and the overarching principle is to assign space that maximizes the chance of success for the faculty member. However, the following recommendations should encourage productive interactions between biomedical engineering and the medical school:

- Long-term planning of space should include opportunities to create space that is co-owned or managed by both engineering and medicine. This might take the form of an interdisciplinary center or institute. This creates regular and long-term interactions between administrative leaders and faculty researchers in both engineering and medicine.
- As described above, assigning research space should be linked to the flow of indirect costs. Care should be taken to understand this dynamic at each university.

### Events or Activities to Stimulate Interaction

While random interactions between faculty in biomedical engineering and the medical school will occur, there are a host of events or activities that can significantly increase these productive interactions. Below is a partial list:

- Establish a “Collaboration Grant” program between biomedical engineering and the medical school. At a minimum these seed grants should be large enough to support a trainee and supplies for a year, and the investigators should be held accountable for progress. A useful endpoint is to require a plan to submit a larger grant at the end of the seed grant period. Both engineering and medicine should provide equal resources for such a program.
- Host a joint thematic research symposia or research retreat that capitalizes on existing strengths or encourages the creation of a new area of research.
- Minimize effort to commute between engineering and medicine. Lowering this barrier (e.g., provide free parking, frequent shuttles) is surprisingly important to increase the frequency, and thus success, of interactions.
- Develop specific activities that encourage biomedical engineering students to be at the medical school. Successful methods include a large number of potential thesis advisors for doctoral students, funding *via* training grants, and undergraduate design teams who are required to develop a project at the medical school.
- Invite chairs of medical school departments to visit the department of biomedical engineering. This is quite simple and can take the form of a research seminar, or a visit during a faculty meeting where the medical school chair and biomedical engineering chair have an opportunity to provide an overview of department interests and priorities.
- Encourage the formation of research centers and/or institutes with themes that engage both engineering and medicine (e.g., systems biology) at a scientific level, and have shared leadership between the schools. Indirect cost recovery of the center should not compete with the department, and if the center has a formal training or educational mission, care must be taken such that it does not conflict or compete with pre-existing programs at the departmental level.

## Summary and Conclusion

It is clear that the educational, translational, and scientific impact of a department of biomedical engineering can be significantly enhanced by close interactions and collaboration with a medical school. However, significant obstacles exist that must be addressed to maximize this opportunity. For example, the academic culture of engineering is strongly influenced by undergraduate education, which does not exist at medical schools. In contrast, a medical school has tremendous pressure to generate clinical income, which can deter research collaborations. Nonetheless, we have outlined recommendations that can facilitate the creation and maintenance of a productive and synergistic relationship between a biomedical engineering department and a medical school. The guidelines provided in this paper are intended to improve these interactions.

